# "Echocardiography in Nigeria: use, problems, reproducibility and potentials"

**DOI:** 10.1186/1476-7120-4-13

**Published:** 2006-03-21

**Authors:** Okechukwu S Ogah, Ademola T Adebanjo, Adedeji S Otukoya, Tarwanger J Jagusa

**Affiliations:** 1 Department of Medicine, Federal Medical Centre, Idi-Aba, PMB 3031 Abeokuta, Nigeria; 2 Department of Family Medicine, Federal Medical Centre, Idi-Aba, PMB 3031 Abeokuta, Nigeria

## Abstract

**Background:**

Although echocardiography is a useful and cost-effective technique for the detection of morphological and functional cardiac abnormalities, it has a main limitation in its subjectivity. Therefore the aim of the present study was to assess the intra-observer reproducibility and validity of 2-dimensional guided M-mode echo measurements at a Nigerian metropolitan Hospital

**Methods:**

Standard echocardiographic examination was performed on twenty randomly selected patients (11 men and 9 women) aged 59.8 ± 12.6 years in two different sessions seven days apart.

**Results:**

A good degree of intraobserver agreement was observed between test 1 and test 2. The correlation coefficient between the first and second studies ranged between 0.60 and 0.96; measurement errors between 0.050 and 0.205.

**Conclusion:**

We would conclude that 2-dimensional guided M-mode measurements at echocardiography performed at our centre are reproducible with low intra-observer variability.

## Background

Echocardiography came into use in Nigeria in the mid 70's, however, in very few centres mostly concentrated in urban areas. Accessibility to echocardiography in Nigeria is still very low due to the high costs of the technique and to the lack of highly specialized personnel performing it. In fact, the country has less than fifty cardiologists serving a population of over 120 million inhabitants. Training in echocardiography is part of the postgraduate residency training requirements in cardiology in Nigeria.

The country, like most developing nations does not have an accreditation process as those designed in Europe and the United States. Separate proficiency examinations in echocardiography such as the British Society of echocardiography examination or similar examination recently introduced by the European Society of Echocardiography are not available. Nevertheless, some of the cardiologists had part or all of their clinical training in advanced countries.

Echocardiography is a highly subjective technique that requires a standardized approach due to its proven clinical usefulness.

Therefore, the aim of our study was to assess the intra-observer variability in M-mode echocardiographic measurements in an urban hospital and to provide a picture of the main indications to an echo exam in Nigeria. This type of survey was never addressed before. We therefore, assessed the intraobserver variability in echocardiographic measurement in our centre.

## Methods

The study was carried out at the Department of Medicine, Federal Medical Centre, Abeokuta, Nigeria between September and November 2005. The centre is a relatively young tertiary institution, established in 1993 by the Federal Government of Nigeria to cater for the health need of the people of Ogun State in South-western Nigeria. The state has a population of about 3.2 million and a land area of about 16,409.26 square kilometres.

Echocardiography is performed at our centre on a weekly basis except in emergency situations. An average of ten echocardiograms is performed per week.

During the period of the study, a total of one hundred and four subjects had echocardiogram. The main indications for echocardiography were hypertensive heart disease, congestive cardiac failure, heart murmur and pre-operative evaluation of cardiac function. Twenty-three of the subjects were randomly selected for the study. (with the use of computer generated random numbers) Two of the subjects had poor 'echo' window and one had regional wall motion abnormality due to left bundle branch block and were therefore excluded. All the other twenty subjects had symmetrically contracting left ventricles.

Informed consent was obtained from the subjects and ethical approval was obtained from our institution's ethical review committee.

### Clinical evaluation

Baseline clinical and demographic characteristics were obtained from the subjects. These included date of birth, age, gender and indication for echocardiogram.

Blood pressure measurements were obtained according to standard guidelines [1] with a mercury sphygmomanometer (Accosson London). Systolic and diastolic blood pressures were measured at Korotkoff sounds phase I and V respectively. Blood pressure was measured at the right arm three times after a 5 minutes rest. Blood pressure 140/90 and above was taken as hypertension. Subjects were weighed without shoes and in light clothing on a standard beam balance. Height was measured to the nearest centimetre using anthropometrical plane with subjects not putting on shoes or headgear.

Body mass index (BMI) was calculated using the formula: BMI = Weight (kg)/Height^2^(m^2^). Body surface area (BSA) was calculated using the formula of Dubois [2].

### Echocardiography

Two-dimensional guided M-mode echocardiography with the use of commercially available echo-machine (ALOKA SSD-1, 100) and a 3.5 MHz linear array transducer was performed on each subject in the partial decubitus position. All measurements were made according to the American Society of Echocardiography (ASE) leading edge to leading edge convention [3]. LV measurement was obtained at end diastole and end systole in the parasternal long axis view. The LV measurements taken include right ventricular outflow tract diameter (RVOT), aortic root diameter (AO), and aortic valve opening (AVO) and left atrial diameter (LA). Others include interventricular septal thickness at end-diastole (IVSTd) and end-systole (IVSTs), the posterior wall thickness at end diastole (PWTd) and end-systole (PWTs), and the LV internal dimensions at end systole (LVIDs) and end diastole (LVIDd). The end of diastole was taken as the peak of the R-wave of the ECG tracing on the echocardiograph while the end-systolic measurements were taken at the nadir of the LV septal wall [3].

LV mass was calculated from the ASE measurements using the cubed formula [4]:

LV mass (g) = 0.8 × {1.04 × [(IVSTd + LVIDd + PWTd)^3 ^- (LVIDd)^3^]} + 0.6

All the measurements were taken at baseline and one week after. Measurements were taken online and in three cardiac cycles and average of the three values calculated. Off-line measurements were not possible because of the lack of the software in our centre. Image storage was with the use of videotapes. One experienced cardiologist performed all the echocardiography.

### Data analysis

Data management and analysis were performed with SPSS software version 11.0. (SPSS, Inc. Chicago, Illinois). Continuous variables were expressed as mean ± SD (standard deviation) and categorical variables expressed as percentages. The paired student t-tests was used to compare the means of baseline and repeated measures. Differences between the repeated measures (measurement 1 minus measurement 2) were plotted against the mean of repeated measures (measurement 1 plus measurement 2 divided by 2) according to the method of Bland and Altman[5]

A 2-tailed p value <0.05 was considered to be significant.

## Results

Table1 shows the baseline clinical and demographic characteristics of the subjects. The study subjects were made up of eleven men and nine women constituting 55% and 45% respectively. The mean age was 59.8± 12.6 years (range 39–76 years).

**Table 1:** Baseline characteristics of the subjects

Age	59.8 (12.6)
Gender (Male/Female)	11/9(55%/45%)
Weight (Kg)	73 (19.3)
Height (cm)	161.5 (9.4)
Body Mass Index (Kg/m^2^)	28.2 (6.9)
Body Surface Area (m^2^)	1.77 (0.25)
Systolic Blood Pressure (mmHg)	139.4 (26.6)
Diastolic Blood Pressure (mmHg)	84.7 (16.5)
Heart Rate (beats/min)	85.6 (12.9)
Indication for Echocardiography	
• Hypertensive Heart Disease	16(80%)
• Rheumatic Heart Disease	2(10%)
• Dilated Cardiomyopathy	2(10%)

The mean body mass index was 28.2 ± 6.9 kg/m^2 ^while mean body surface area was 1.77 ± 0.25 m^2^. Mean systolic blood pressure, diastolic blood pressure and pulse rate were 139.4 ± 26.6 mmHg, 84.7 ±16.5 mmHg and 85.6 ± 12.9 beats/min respectively.

The indications for echocardiography were hypertensive heart disease (sixteen subjects), rheumatic heart disease (two subjects) and dilated cardiomyopathy (two subjects).

Echocardiographic diagnosis made were hypertensive heart disease (left ventricular hypertrophy and/or diastolic dysfunction) in ten of the sixteen hypertensives, and normal study in the remaining six subjects. Mixed mitral valve disease (but predominantly mitral stenosis) was diagnosed in the two female subjects with rheumatic heart disease.

The dilated cardiomyopathy cases were pregnancy related (peripartal cardiomyopathy) which is a common disorder in Nigeria especially in northern Nigeria, which is reported to have the highest incidence in the world.

**Figure 1:**
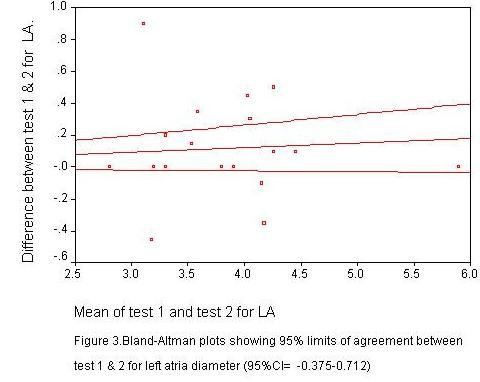
Bland-Altman plots showing 95% limits of agreement between test 1 and 2 for left atrial diameter (95%CI = -0.375-0.712)

**Figure 2:**
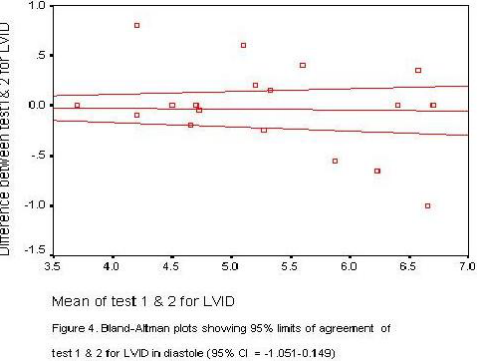
Bland-Altman plots showing 95% limits of agreement between test 1 and test 2 for LVID in diastole (95% CI = -1.051-0.149)

Scatter plots of LV measurements were performed for measurements 1 and 2 (plots not shown) Bland – Altman plots were also performed to determine the 95% confidence limits of agreement between the two measurements. Two of such plots are shown in figures 1 and 2.

The plots provided visual information on the degree of disagreement, as well as the relationship of the differences and size of the measurements. Most of the plots were near the zero- difference line and showed uniform distribution pattern.

Table 2 displays the degree of correlation between the first and second measurements as well as the 95% confidence intervals. There was good correlation between the two measurements in all the parameters. The intraobserver concordance correlation coefficient for RVOT, aortic root diameter, aortic valve separation, left atrial diameter, IVSd, IVSs, LVID, LVIS, PWTd PWTs and LVM were 0.76, 0.91, 0.60, 0.91, 0.93, 0.81, 0.92, 0.96, 0.75, 0.79 and 0.89 respectively.

**Table 2:** Correlation between the two measurements

**PARAMETER**	**r**	**P**	**95% CI**
RVOT	0.76	<0.0001	0.470–898
AO	0.91	<0.0001	0.787–0.965
AVO	0.60	0.0041	0.217–0.825
LA	0.91	<0.0001	0.789–0.970
IVSD	0.93	<0.0001	0.835–0.933
IVSS	0.81	<0.0001	0.566–0.920
LVID	0.92	<0.0001	0.795–0.967
LVIS	0.96	<0.0001	0.90–0.98
PWTd	0.75	<0.0001	0.453–0.894
PWTs	0.79	<0.0001	0.526–0.911
LVM	0.89	<0.0001	0.748–0.958

RVOT = Right Ventricular Outflow Diameter, AO = Aortic Root Diameter, AVO = Aortic Valve Opening, LA = Left Atrial Diameter, IVSd = Interventricular Septal wall thickness in diastole, IVSs = Interventricular septal wall thickness in systole, LVIDs = Left ventricular Internal Diameter in Diastole, LVIS = Left Ventricular internal diameter in Systole, PWTd = Posterior Wall Thickness in Diastole, PWTs = Posterior Wall Thickness in Systole, LVM = Left Ventricular Mass

Table 3 depicts the intraobserver comparisons. Difference between first and second measurement of interventricular septal wall thickness in diastole was statistically significant.

**Table 3:** Intraobserver comparison of Echocardiographic variables using paired t Test

PARAMETER	n	TEST 1(SD)	TEST 2 (2D)	MEAN DIFFERENCE (2D)	P VALUE
RVOT	20	2.95 (0.58)	2.99(0.53)	-0.06(0.39)	0.534
AO	20	2.74(0.36)	2.77 (0.36)	-0.025(0.152)	0.470
AVO	20	1.86 (0.46)	1.94 (0.35)	-0.08 (0.37)	0.365
LA	20	3.89(0.69)	3.76(0.71)	0.12 (0.29)	0.078
IVSd	20	1.02 (0.27)	0.93(0.25)	0.09 (0.10)	0.001
IVSs	20	1.26(0.36)	1.22(0.34)	0.04 (0.22)	0.450
LVIDd	20	5.33(0.88)	5.35 (1.08)	0.02 (0.41)	0.850
LVIDs	20	3.80(1.08)	3.83 (1.24)	0.03 (0.37)	0.744
PWTd	20	1.08 (0.20)	1.10 (0.23)	0.03(0.16)	0.744
PWTs	20	1.46 (0.25)	1.56(0.29)	0.10 (0.18)	0.575
LVM	20	217.5(70.2)	211.8(81.0)	5.68(36.3)	0.492

RVOT = Right Ventricular Outflow Diameter, AO = Aortic Root Diameter, AVO = Aortic Valve Opening, LA = Left Atrial Diameter, IVSd = Interventricular Septal wall thickness in diastole, IVSs = Interventricular septal wall thickness in systole, LVIDs = Left ventricular Internal Diameter in Diastole, LVIS = Left Ventricular internal diameter in Systole, PWTd = Posterior Wall Thickness in Diastole, PWTs = Posterior Wall Thickness in Systole, LVM = Left Ventricular Mass

Large standard deviations of the difference were found for end diastolic LV diameter, right ventricular outflow tract diameter, aortic root diameter, aortic valve opening and LVM.

## Discussion

Echocardiography is becoming a common practice in Nigeria especially in the major cities. Conventional echocardiographs are commoner. Portable echo-machine is probably available in only one centre. Transoesophageal echocardiography is yet to be introduced into the country. Measurements are obtained on-line in all the centres due to the high cost of off-line software and machines. Recording is mainly by videotapes.

The common indications for echocardiography in Nigeria are hypertensive heart disease, cardiomyopathies, and valvular heart disease.

In a report by Balogun et al[6], hypertensive heart disease, cardiomyopathies, normal echocardiogram, valvular heart disease and pericardial diseases constituted 53%, 21%, 13%, 7%, and 4% respectively of echocardiographic diagnoses in their series. An audit of 1544 echocardiograms performed over a 19-month period (article in press) showed that hypertensive heart disease (51.8%), normal study (36.5%), valvular heart disease (3.5%), dilated cardiomyopathy (1.9%) were the most frequent diagnoses.

The potential of echocardiography as a research tool in Nigeria cannot be overemphasized. Studies emanating from the country have focused on the common cardiovascular diseases in the country such as hypertensive heart disease [7-15], heart failure[16,17], dilated cardiomyopathy including peripartal heart disease, and valvular heart disease[18,19]. Others have also studied cardiac function in diabetes mellitus[20-22], congenital heart diseases[23] and sickle cell disease[24]. The usefulness of ECG criteria for the diagnosis of left ventricular hypertrophy in Nigerians using echocardiography as standard has also been reported [25].

This quality assessment study addressed the intraobserver variation of 2-dimensional guided M-mode echocardiographic measurements in our centre. The study demonstrated that echocardiographic measurements by a single cardiologist is consistent and has acceptable intraobserver variation. Interobserver variation was not evaluated in this study because only one cardiologist performs the procedure in our hospital.

Results from other studies on intraobserver variations of echocardiographic measurements vary in study design and method of analyses.

Schieken et al [26] studied the intraobserver variability of aortic root diameter, left atria diameter, LV septal and posterior wall thickness, LV interval dimensions and ejection time in 20 healthy children aged 6–16 years. The measurement errors (standard deviation divided by 2) were reported as 0.5, 0.6, 0.6, 1.3, 0.6, 1.0 mm and 0.01 second respectively. These are comparable to the findings in this study (Table 3). Our measurement errors were 0.195, 0.076, 0.185, 0.145, 0.050, 0.110, 0.202, 0.185, 0.080, and 0.090 for RVOT, aortic root diameter, aortic root separation, left atrial diameter, IVSd, IVSs, LVID, LVIS, PWTD, and PWTs respectively. Our finding is similar to the observation of Dai et al[27]. Valdez et al[28] reported significant intraobserver difference in only one person in the measurement of LV end diastolic posterior wall diameter in his study of 20 subjects. In the present study significant intraobserver difference was observed only in LV septal wall thickness in diastole (LVIDd).

In the study by Ladipo et al [29], intraobserver variations were assessed in 10 subjects. The mean difference of 0.7–1.2, 0.2–0.8, 0.3–0.4 and 0.4–0.8 mm were reported for LV internal dimensions in diastole and systole, LV posterior wall thickness in diastole, LV septal wall thickness in diastole respectively. Table 4 shows a summary of previous studies and present study.

**Table 4:** Published studies and Present study: Intraobserver Comparison of Echocardiographic Measurements

	**Dai et al (ref 27)**			**Present Study**			**Ladipo et al (ref 29)**			**Schieken et al (ref 26)**		
Parameter	Mdiff	SD	Error	Mdiff	SD	Error	Mdiff	SD	Error	Mdiff	SD	Error
RVOT	NA	NA	NA	-0.06	0.39	0.195	NA	NA	NA	NA	NA	NA
AO	0.05	1.20	0.60	0.025	0.152	0.076	NA	NA	NA	NA	NA	0.05
AVO	NA	NA	NA	-0.08	0.37	0.185	NA	NA	NA	NA	NA	NA
LA	0.15	1.35	0.675	0.12	0.29	0.145	NA	NA	NA	NA	NA	0.06
IVSd	0.05	0.82	0.41	0.09	0.10	0.05	0.04–0.08	0.06–0.18	0.03–0.09	NA	NA	0.06
IVSs	0.05	1.13	0.565	0.04	0.22	0.11	NA	NA	NA	NA	NA	NA
LVIDd	-0.60	0.97	0.485	0.02	0.41	0.205	0.07–0.12	0.10–0.28	0.05–0.14	NA	NA	0.13
LVIDs	-0.15	1.18	0.595	0.03	0.37	0.185	0.02–0.08	0.09–0.20	0.05–0.10	NA	NA	0.10
PWTd	0.13	0.84	0.42	0.03	0.16	0.080	0.06–0.10	0.09–0.27	0.05–0.14	NA	NA	0.06
PWTs	0.33	1.14	0.57	0.10	0.18	0.090	NA	NA	NA	NA	NA	NA
LVM	-1.82	18.79	9.40	5.68	36.3	18.1	NA	NA	NA	NA	NA	NA

RVOT = Right Ventricular Outflow Diameter, AO = Aortic Root Diameter, AVO = Aortic Valve Opening, LA = Left Atrial Diameter, IVSd = Interventricular Septal wall thickness in diastole, IVSs = Interventricular septal wall thickness in systole, LVIDs = Left ventricular Internal Diameter in Diastole, LVIS = Left Ventricular internal diameter in Systole, PWTd = Posterior Wall Thickness in Diastole, PWTs = Posterior Wall Thickness in Systole, LVM = Left Ventricular Mass, Mdiff = Mean difference, SD = Standard Deviation, Error = SD/2 NA = Not Available.

The main limitation of echocardiography is its subjectivity in the face of a proven clinical usefulness. Training and accreditation procedures are key factors to obtain a reliable and standard examination. The results of our study demonstrate that when the same person with an appropriate training performs echocardiography, the intra-observer variability is low. This assessment is important in order to have an internal control for our laboratory since this technology might represent the only possible procedure for any given clinical condition. Our data are consistent with previous reports.

Workers have highlighted the sources of variation in echocardiographic measurements[30-32]. These include factors that affect image quality such as subject's body build, respiratory status and co-operation. Others include ability of the sonographer to correctly recognize image signals, transducer orientation and placement as well as his/her familiarity with the machine.

### Limitations of the study

Interobserver variability was not analyzed in the present study since echocardiography is performed at our center by only one cardiologist. In Nigeria as previously stated, the number of cardiologists is very small. Nonetheless this analysis and results were aimed at providing an objective assessment of the quality of our laboratory. Furthermore, Doppler measurements were not evaluated. We also did not obtain measurement using the 2D technique, which has been shown to have better reliability than the M-mode technique

## Conclusion

We would conclude that quantitative 2-dimensional guided M-mode echocardiography performed in our centre is reproducible and has good validity. It therefore provides a valuable tool for cardiac structure and function studies.

## Competing interests

The authors declare that they have no competing interests.

## Authors' contributions

OSO conceived of the study, carried out the echocardiograms, analysed the data and drafted the manuscript.

AAT, OAS and JJT participated in the recruitment of subjects and in data management. All authors read and approved the final manuscript
